# Impact of Moderna mRNA-1273 Booster Vaccine on Fully Vaccinated High-Risk Chronic Dialysis Patients after Loss of Humoral Response

**DOI:** 10.3390/vaccines10040585

**Published:** 2022-04-11

**Authors:** Sammy Patyna, Timon Eckes, Benjamin F. Koch, Stephan Sudowe, Anke Oftring, Niko Kohmer, Holger F. Rabenau, Sandra Ciesek, Despina Avaniadi, Rahel Steiner, Ingeborg A. Hauser, Josef M. Pfeilschifter, Christoph Betz

**Affiliations:** 1 Division of Nephrology, Department of Internal Medicine III, University Hospital Frankfurt, Goethe University Frankfurt, 60590 Frankfurt, Germany; benjamin.koch@kgu.de (B.F.K.); despina.avaniadi@kgu.de (D.A.); rahel.steiner@kgu.de (R.S.); ingeborg.hauser@kgu.de (I.A.H.); christoph.betz@kgu.de (C.B.); 2 Institute of General Pharmacology and Toxicology, University Hospital Frankfurt, Goethe University Frankfurt, 60590 Frankfurt, Germany; eckes@em.uni-frankfurt.de (T.E.); oftring@med.uni-frankfurt.de (A.O.); pfeilschifter@em.uni-frankfurt.de (J.M.P.); 3 GANZIMMUN Diagnostics AG Mainz, 55128 Mainz, Germany; dr.sudowe@ganzimmun.de; 4 Institute for Medical Virology, University Hospital Frankfurt, Goethe University Frankfurt, 60590 Frankfurt, Germany; niko.kohmer@kgu.de (N.K.); rabenau@em.uni-frankfurt.de (H.F.R.); sandra.ciesek@kgu.de (S.C.); 5 German Centre for Infection Research, External Partner Site, 60323 Frankfurt, Germany; 6 Fraunhofer Institute for Molecular Biology and Applied Ecology (IME), Branch Translational Medicine and Pharmacology, 60596 Frankfurt, Germany

**Keywords:** COVID-19, SARS-CoV-2, vaccination, hemodialysis, booster, mRNA-1273, seroconversion, T cell response

## Abstract

The long-term effect of protection by two doses of SARS-CoV-2 vaccination in patients receiving chronic intermittent hemodialysis (CIHD) is an urging question. We investigated the humoral and cellular immune response of 42 CIHD patients who had received two doses of SARS-CoV-2 vaccine, and again after a booster vaccine with mRNA-1273 six months later. We measured antibody levels and SARS-CoV-2-specific surrogate neutralizing antibodies (SNA). Functional T cell immune response to vaccination was assessed by quantifying interferon-γ (IFN-γ) and IL-2 secreting T cells specific for SARS-CoV-2 using an ELISpot assay. Our data reveal a moderate immune response after the second dose of vaccination, with significantly decreasing SARS-CoV-2-specific antibody levels and less than half of the study group showed neutralizing antibodies six months afterwards. Booster vaccines increased the humoral response dramatically and led to a response rate of 89.2% for antibody levels and a response rate of 94.6% for SNA. Measurement in a no response/low response (NR/LR) subgroup of our cohort, which differed from the whole group in age and rate of immunosuppressive drugs, indicated failure of a corresponding T cell response after the booster vaccine. We strongly argue in favor of a regular testing of surrogate neutralizing antibodies and consecutive booster vaccinations for CIHD patients to provide a stronger and persistent immunity.

## 1. Introduction

The current coronavirus disease 2019 (COVID-19) pandemic confronts our society with a global threat that particularly concerns patients with compromised immune systems, either due to immunosuppressive therapy or diseases associated with impaired immune response. Patients with end-stage renal disease (ESRD) undergoing chronic intermittent hemodialysis (CIHD) are affected by lower humoral and cellular immunity [1]. For this reason, severe acute respiratory syndrome coronavirus-2 (SARS-CoV-2) infection and chronic dialysis patients make a particular dangerous relationship. Patients undergoing CIHD represent a vulnerable population which are at an increased risk of SARS-CoV-2 infection and threatened by an almost four-fold higher mortality rate compared to the general population [2,3]. Thus, CIHD patients were prioritized for early SARS-CoV-2-vaccination in several countries [4]. Sahin et al. showed that vaccination with BNT162b2 (Tozinameran, BioNTech/Pfizer, Mainz, Germany/New York, NY, USA) induced both a strong antibody and T cell response in healthy adults [5,6]. However, recent data reveal a significantly lower SARS-CoV-2 spike protein antibody titer and reduced vaccination success rates of patients receiving CIHD compared to the general population after vaccination with BNT162b2 [7]. An effective short-term seroconversion rate after SARS-CoV-2 vaccination in the CIHD population was shown but recently published results indicate rapidly decreasing SARS-CoV-2-specific antibody levels in the long term [8]. There is evidence for a beneficial effect of a third dose of a COVID-19 mRNA vaccine on antibody titers in this specific high-risk population [9] and, in addition to the humoral immune response, T cells may also contribute to a protective immunity against SARS-CoV-2 and improve vaccine efficacy [10,11]. However, data regarding cellular immune response in this cohort are scarce.

Here, we investigated the initial SARS-CoV-2-specific serological and functional T cell immune response after two doses of SARS-CoV-2 vaccination (either BNT162b2 or ChAdOx1 (AstraZeneca, Cambridge, UK)) and its longevity during a time course of up to six months after the second dose in a cohort of high-risk CIHD patients in a tertiary care dialysis unit. In addition, we analyzed antibody titers and surrogate neutralizing antibodies (SNA) after a third dose, often referred to as booster vaccination, with Spikevax (mRNA-1273, Moderna, Cambridge, MA, USA) and measured its effect on the SARS-CoV-2-reactive T cell response in a subgroup of CIHD patients with no response/low response (NR/LR) (*n* = 10) after their second vaccination.

## 2. Materials and Methods

### 2.1. Study Design and Cohorts

In a prospective, non-interventional study approach, 42 long-term dialysis patients were observed from January 2021 to December 2021 in a tertiary care dialysis unit at the Department of Nephrology and Dialysis at Frankfurt University Hospital (Frankfurt, Germany). A healthy control group (*n* = 11; median (IQR) age 34 years (26–49), 27.3% male) was included to compare cellular immune responses after immunization with two doses of BNT162b2 (BioNTech/Pfizer) mRNA vaccine.

**Figure 1:**
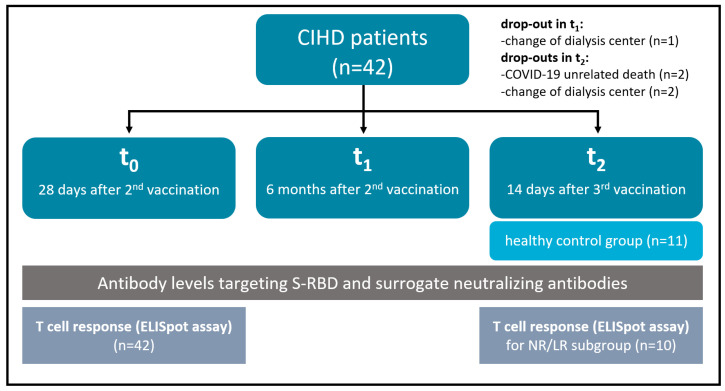
Illustration of the study setup. Samples were collected at three different time points to evaluate the dynamic of the patients’ immunization status by different immunoassays. Abbreviations: CIHD (chronic intermittent hemodialysis); S-RBD (spike receptor binding domain); ELISpot (enzyme-linked immunospot).

Antibody levels and SNA were measured four weeks after the second dose (=t_0_, either with BNT162b2 or ChAdOx1), six months after second dose (=t_1_) and 14 days after a third dose (=t_2_, booster vaccine) of mRNA-1273 (Moderna) (Figure 1), exploring the time course of SARS-CoV-2-specific antibody levels and the percentage of neutralizing antibodies after vaccination against SARS-CoV-2 during the COVID-19 pandemic.

The study was conducted in accordance with the principles of the Declaration of Helsinki and approved by the local ethics committee (reference number: 20/658). All study participants provided written informed consent before study entry.

### 2.2. Assessment of SARS-CoV-2-Specific Antibodies 

Levels of antibodies targeted against SARS-CoV-2 spike receptor binding domain (S-RBD) were measured with a chemiluminescent microparticle based immunoassay (SARS-CoV-2 IgG II Quant, Abbott GmbH, Wiesbaden, Germany). Results are expressed as standardized binding antibody units (BAU)/mL. According to the manufacturer, a cut-off for positivity is set to 7.1 BAU/mL. We elevated the cut-off to 8.52 BAU/mL by adding a “threshold” range in between.

### 2.3. Assessment of SARS-CoV-2-Specific Surrogate Neutralizing Antibodies (SNA)

The binding of S-RBD to angiotensin-converting enzyme 2 allowing detection of SARS-CoV-2-neutralizing antibodies was performed using the ELISA-based GenScript SARS-CoV-2 Surrogate Virus Neutralization Test Kit (GenScript Biotech, Piscataway Township, NJ, USA) [12]. Results are expressed as percentage inhibition (%-INH). 

### 2.4. Assessment of Cytokine-Producing SARS-CoV-2-Reactive T Cell Responses

The adaptive immune response was analyzed by quantifying interferon-γ (IFN-γ) and IL-2 secreting T cells specific for SARS-CoV-2 using a multicolor fluorescence enzyme-linked immunospot (ELISpot) assay (CoV-iSpot, AID GmbH, Strassberg, Germany) [13]. Highly specific sequences of immunodominant epitopes of SARS-CoV-2 structural proteins (=SARS-CoV-2-peptide-mix) were used for stimulation of T cells. Results are expressed as spot forming cells (SFC)/10^6^ lymphocytes.

### 2.5. Statistics

Continuous variables are shown as median and interquartile range (IQR), categorical variables are reported as frequencies and percentages. Differences between patient cohorts were determined using the Fisher’s exact test for categorical variables; for quantitative variables, a *t*-test or a Mann–Whitney-U test was used for parametric and nonparametric data, respectively. When comparing more than two groups, one-way ANOVA followed by Bonferroni’s post hoc correction or Kruskal–Wallis test followed by Dunn´s post hoc test was performed for parametric and nonparametric data, respectively. For the comparison of related samples comprising more than two groups, a Friedman test was performed. Spearman´s rank correlation analysis was used to determine bivariate relationships by calculating Spearman´s Rho (correlation coefficient). Normality was assessed by a Kolmogorov–Smirnov test. All *p*-values reported are two-sided, the level of significance was set *p* < 0.05. Statistical analyses were performed using GraphPad Prism version 9.3.1 (GraphPad Software, San Diego, CA, USA) and BiAS (v11.01; epsilon-Verlag, Nordhasted, Germany).

## 3. Results

### 3.1. Basic Study Cohort Characteristics

We prospectively enrolled 45 CIHD patients, of which 42 met inclusion criteria, with a median (IQR) age of 62 (52–72.5) years. Three patients were excluded due to SARS-CoV-2 positivity (real-time reverse transcriptase PCR). The main clinical and demographic characteristics are depicted in Table 1. Due to the incidence of comorbidities, this study cohort is representing a highly vulnerable CIHD group. Five patients were not available for follow-up because of death unrelated to COVID-19 (*n* = 2, sepsis and cardiac arrest) and change of the dialysis center (*n* = 3). We observed a good vaccine tolerability, without serious adverse events in this cohort. No patient developed critical side effects or was hospitalized.

### 3.2. Dynamics of Antibody Levels in Vaccinated Dialysis Patients

**Figure 2:**
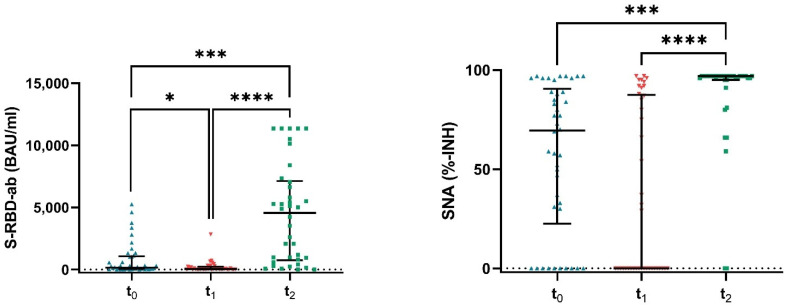
Antibody response in plasma samples of patients taken at three different time points (t_0_ = four weeks after second dose, t_1_ = six months after second dose and t_2_ = 14 days after third dose (=booster vaccine) of mRNA-1273 (Moderna)). Abbreviations: S-RBD-ab (spike receptor binding domain-antibodies); SNA (surrogate neutralizing antibodies). Data are expressed as median (IQR). * *p* ≤ 0.05, *** *p* ≤ 0.001, **** *p* ≤ 0.0001 (Friedman test).

Four weeks after the second dose of vaccination (=t_0_), 37 patients (88.1%) developed antibodies against the SARS-CoV-2 spike protein with a median (IQR) of 128 BAU/mL (43–1027.5), and 32 patients (76.2%) showed functional neutralizing capacity of SNA with a median (IQR) of 69%-INH (15–89), above the predefined threshold for positivity (>8.52 BAU/mL and >30%-INH, respectively; Figure 2).

During the six-month period, the response rate of positive serology did not change (87.8%) but antibody levels decreased significantly compared to t_0_ (median (IQR) of 32.3 BAU/mL (9.9–224.1), *p* < 0.014). Less than half of this study group showed neutralizing antibodies (48.8%) with a median (IQR) of 0%-INH (0–87.8) six months after the second dose of the vaccine. Measuring antibody levels and SNA after the booster vaccine (=t_2_), we observed a significant elevation compared to timepoint t_0_ (*p* < 0.001) and t_1_ (*p* < 0.001) with a response rate of 89.2% for antibody levels (median (IQR): 4560 BAU/mL (646.7–7272.5)) and a response rate of 94.6% for SNA (median (IQR) of 97%-INH (0–97)). This corresponds to a 36-fold increase in median antibody titers after the third dose compared to t_0_. 

**Figure 3:**
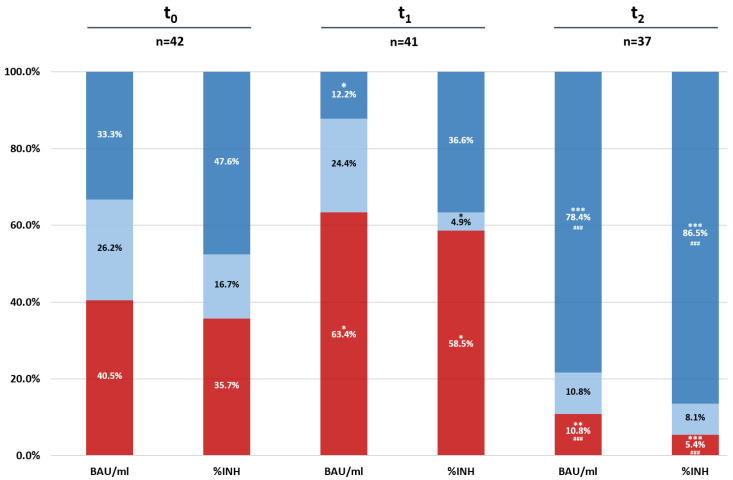
Proportion of patients with strong (dark blue: >500 BAU/mL or >70%-INH), moderate (light blue: 100–500 BAU/mL or 50–70%-INH) and no/low (red: <100 BAU/mL or <50%-INH) humoral response at the three different time points (t_0_, t_1_ and t_2_). Data are expressed as percentage. * *p* ≤ 0.05, ** *p* ≤ 0.01, *** *p* ≤ 0.001 compared to t_0_; ^###^ *p* ≤ 0.001 compared to t_1_.

To get a more detailed insight into the distribution of the humoral immune response we predefined different threshold values depending on the level of antibodies or neutralization capacity. Applying these antibodies and SNA cut-off levels (<100 BAU/mL or <50%-INH, no response/low response; 100–500 BAU/mL or 50–70%-IHN, moderate response; >500 BAU/mL or >70%-IHN, strong response) the overall response rates at t_0_, t_1_ and t_2_ are described in Figure 3, demonstrating a significant reduction in the humoral immunity six months after the second dose of the vaccine (=t_1_), followed by a distinct increase two weeks after the booster vaccine (=t_2_) with a significant difference, respectively. 

**Figure 4:**
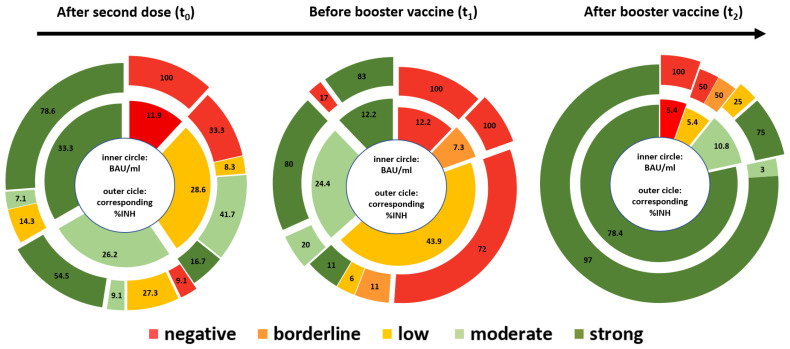
Fraction of neutralizing capacity for different antibody levels. The inner circle is showing levels of antibody response (BAU/mL) with the corresponding percentage of neutralizing antibodies (%-INH) in the outer circle for t_0_, t_1_ and t_2_, respectively. Data are expressed as percentages. Applying the following antibody and SNA cut-off levels: negative (<7.1 BAU/mL or <30%-INH), borderline (>7.1–8.5 BAU/mL or >30–35%-IHN), low (>8.5–100 BAU/mL or >35–50%-INH), moderate (>100–500 BAU/mL or >50–70%-INH), strong (>500 BAU/mL or >70%-INH).

The neutralizing capacity of SARS-CoV-2-specific antibodies is even more important than only the magnitude of antibody levels. For this reason, we analyzed the fraction of neutralizing capacity for different antibody levels, as illustrated in Figure 4. Data from our study group reveal that levels of SARS-CoV-2-spike-specific antibodies are significantly correlated with the percentage of SNA (t_0_ rho: 0.83; *p* < 0.001; t_1_ rho: 0.9; *p* < 0.001 and t_2_ rho: 0.58; *p* < 0.001).

### 3.3. T Cell Response after Vaccination 

**Figure 5:**
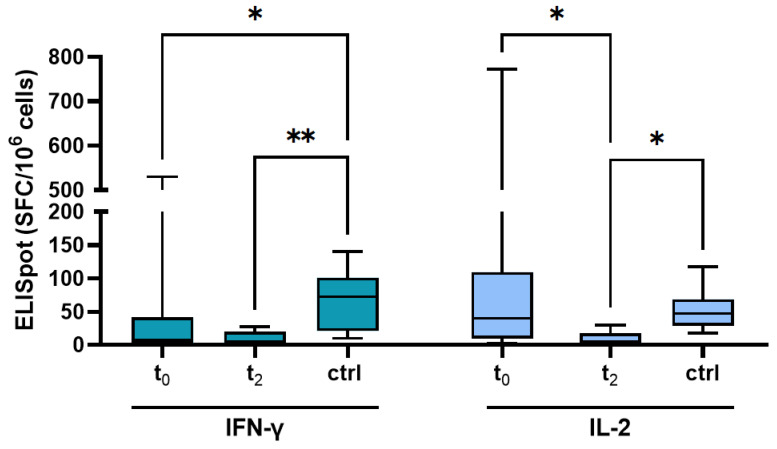
Cytokine profile of T cell response specific to SARS-CoV-2 at different time points and in comparison to a healthy control group. Abbreviations: ELISpot (enzyme-linked immunospot); ctrl (control); IFN-γ (Interferon-γ); IL-2 (Interleukin-2). Data are expressed as median (IQR). Intragroup paired analysis: * *p* ≤ 0.05, ** *p* ≤ 0.01 as indicated (Kruskal–Wallis test).

Four weeks after the second dose, SARS-CoV-2-specific T cell reactivity of CIHD patients showed significantly lower levels of IFN-γ release compared to the healthy control group (HC, *n* = 11) (median (IQR) 7.5 SFC/10^6^ cells (2.5–42.5) vs. 72.5 SFC/10^6^ cells (21.25–101.25) (*p* < 0.001)), but no difference in IL-2 release between both groups (median (IQR) 40 SFC/10^6^ cells (8.75–111.25) vs. 47.5 SFC/10^6^ cells (28.75–68.75) (*p* < 0.001)); Figure 5. A positive IFN-γ release was only observed for nine patients (21.4%). A positive release of IL-2 after the booster vaccine occurred in 17 patients (40.5%). 

We measured the T cell response again after the booster vaccination (t_2_) in a no response/low response NR/LR subgroup (*n* = 10) of t_0_. One patient showed a positive IFN-γ release after the second dose, but none of the NR/LR group showed a positive release of IL-2. Although data of NR/LR patients present a robust serological response to the booster vaccination (median (IQR): 990 BAU/mL (235.3–5033.3); 94%-INH (64.3–97)), only two patients showed a positive release of IFN-γ and only one patient for IL-2. Compared to the remaining study group (median (IQR): 5261 BAU/mL (970.8–10,238)), we measured significant lower antibody levels in the NR/LR subgroup (*p* < 0.049). In summary, only one patient in the NR/LP subgroup developed a T cell response after booster vaccination (IFN-γ: 132.5 SFC/10^6^ lymphocytes; IL-2: 165 SFC/10^6^ lymphocytes).

## 4. Discussion

Patients undergoing CIHD represent a vulnerable population with an increased risk of SARS-CoV-2 infection, higher disease severity and COVID-19-related mortality risk [14]. For this group, there is still lacking data for long-term humoral and cellular immune responses, especially relating to the presence of neutralizing antibodies. In this study, we analyzed the magnitude and kinetics of antibody and T cell responses to the initial immunization (t_0_), the status quo after 6 months (t_1_) and the state following a booster with Moderna’s mRNA-1273 (t_2_). In our cohort of hemodialysis patients, 88.1% developed antibodies against the SARS-CoV-2 spike protein 4 weeks after the primary vaccine series, and 76.2% showed functional neutralizing capacity of SNA (surrogate neutralizing antibodies). It was reported that neutralizing antibody titers decreased over time in patients after SARS-CoV-2 infection [15]. However, after 6 months we can clearly see a significant decline, not only in antibody levels, but even more decisively, a loss in neutralizing capacity. Importantly, more than half of our patients (51.2%) had no levels of SARS-CoV-2-neutralizing antibodies at that time. Comparable results have been reported by Ducloux et al. and Kohmer et al. for Pfizer-BioNTech’s BNT162b2 [9,16]. In line with our data, both studies confirm that levels of SARS-CoV-2-specific antibodies were significantly increased by administering a third dose of a COVID-19 mRNA vaccine. However, cell-mediated immunity was not analyzed in these studies. 

We conclude that a decline in neutralizing antibody levels could be prevented by a timed booster intervention for our cohort. However, measurement in a NR/LR subgroup of our cohort also indicates failure of a corresponding T cell response after the booster vaccine. These findings are in line with data of Espi et al. that describe a missing increase in T cell response after third dose vaccination in dialysis patients [17]. Notably, the NR/LR subgroup showed a higher rate of immunosuppressive medication (21.4% vs. 60%, *p* = 0.024) and a higher age (median (IQR): 62 (52–72.5) vs. 74.5 (67.3–79.3)) compared to the whole study group, which could be a confounder and an explanation for the diminished T cell response after booster vaccination. To analyze the influence of these possible confounders, a higher number of participants in the NR/LR subgroup would be required, which poses a limitation of this study. 

Our data underline the importance of SNA when screening for the response of patient´s adaptive immunity after SARS-CoV-2 vaccination, especially in this highly vulnerable group of CIHD patients. Hence, CIHD patients are not only particularly threatened by the COVID-19 pandemic because of a more severe course of disease and a higher risk of infection caused by higher frequency of medical consultations but, most notably, by a lower response after the second dose of vaccination, combined with a relevant loss of both serological and specific T cell immunity. 

T cell mediated immunity seems to play an important role in SARS-CoV-2 infection. However, to what extent is not clear. The presented results are highlighting the importance of an early booster vaccination of CIHD patients. 

Further studies are required to generate convincing data for CIHD patients on long-term T cell response after SARS-CoV-2 vaccination. This study only provides data on T cell response after booster vaccination in a rather small subgroup of initial NR/LR patients. In addition, more investigations regarding protection against newly arising variants (e.g., Omicron) are needed.

## 5. Conclusions

In summary, a third dose of SARS-CoV-2 mRNA-1273 vaccine in high-risk CIHD patients increased the magnitude and the neutralizing capacity of SARS-CoV-2-specific antibodies, even in the NR/LR subgroup. The relevance of a repeated booster vaccination after a certain period to enhance immunity against SARS-CoV-2 provides an incent for the next inoculation campaign for these immunocompromised CIHD patients.

## Figures and Tables

**Table 1:** Baseline patient characteristics. NR/LR (no response/low response) group with insufficient humoral response after two doses of SARS-CoV-2 vaccine (t_0_). Abbreviations: m (male); GN (glomerulonephritis); ADPKD (autosomal dominant polycystic kidney disease); Tx (transplant). Data expressed as number (*n*) and percentage (%) or median (med) and interquartile range (IQR), respectively.

		All Patients		NR/LR Group		*p*-Value
		*n* = 42		*n* = 10		
Age (years)	med (IQR)	62	(52–72.5)	74.5	(67.3–79.3)	0.0041
Gender (male)	*n* (%)	29	(69)	6	(60)	0.7109
Vintage on dialysis (years)	med (IQR)	2.4	(1–7.2)	6.2	(0.9–12)	0.3893
Days after 2nd vaccination (days)	med (IQR)	34.5	(30.5–47.8)	34	(29–47)	0.8189
**Ethnicity**						
Caucasian	*n* (%)	31	(73.8)	8	(80)	1.0000
Black	*n* (%)	6	(14.3)	2	(20)	0.6415
Other	*n* (%)	5	(11.9)	/		
**Reason for HD**						
Diabetic nephropathy	*n* (%)	9	(21.4)	3	(30)	0.6792
Hypertensive nephropathy	*n* (%)	4	(9.5)	1	(10)	1.0000
ADPKD	*n* (%)	6	(14.3)	2	(20)	0.6415
GN	*n* (%)	3	(7.1)	1	(10)	1.0000
Amyloidosis	*n* (%)	5	(11.9)	/		
Chronic interstitial nephritis	*n* (%)	6	(14.3)	/		
Nephrosclerosis	*n* (%)	6	(14.3)	/		
Vasculitis	*n* (%)	1	(2.4)	/		
Others/unknown	*n* (%)	2	(4.8)	3	(30)	0.0432
**Comorbidities**						
Diabetes mellitus	*n* (%)	13	(31.0)	6	(60)	0.1424
Lung disease	*n* (%)	5	(11.9)	2	(20)	0.6079
Smoker	*n* (%)	14	(33.3)	3	(30)	1.0000
Cancer	*n* (%)	5	(11.9)	/		
Severe cardiovascular disease	*n* (%)	23	(54.8)	6	(60)	1.0000
Hypertension	*n* (%)	38	(90.5)	9	(90)	1.0000
Kidney or liver-Tx	*n* (%)	6	(14.3)	4	(40)	0.0847
Immunosupression	*n* (%)	9	(21.4)	6	(60)	0.0243

Data: (%) percentage; (med) median; (IQR) interquartile range; m (male); GN (glomerulonephritis), ADPKD (autosomal dominant polycystic kidney disease).
